# High-throughput bioprinting of the nasal epithelium using patient-derived nasal epithelial cells

**DOI:** 10.1088/1758-5090/aced23

**Published:** 2023-08-14

**Authors:** I Deniz Derman, Miji Yeo, Diana Cadena Castaneda, Megan Callender, Mian Horvath, Zengshuo Mo, Ruoyun Xiong, Elizabeth Fleming, Phylip Chen, Mark E Peeples, Karolina Palucka, Julia Oh, Ibrahim T Ozbolat

**Affiliations:** 1 Engineering Science and Mechanics Department, Penn State University, University Park, PA 16802, United States of America; 2 The Huck Institutes of the Life Sciences, Penn State University, University Park, PA 16802, United States of America; 3 The Jackson Laboratory, Farmington, CT 06032, United States of America; 4 Center for Vaccines and Immunity, Abigail Wexner Research Institute at Nationwide Children’s Hospital, Columbus, OH 43205, United States of America; 5 Department of Pediatrics, College of Medicine, The Ohio State University, Columbus, OH 43210, United States of America; 6 Infectious Disease Institute, The Ohio State University, Columbus, OH 43210, United States of America; 7 Biomedical Engineering Department, Penn State University, University Park, PA 16802, United States of America; 8 Materials Research Institute, Penn State University, University Park, PA 16802, United States of America; 9 Cancer Institute, Penn State University, University Park, PA 16802, United States of America; 10 Neurosurgery Department, Penn State University, University Park, PA 16802, United States of America; 11 Department of Medical Oncology, Cukurova University, Adana, Turkey; 12 Biotechnology Research and Application Center, Cukurova University, Adana, Turkey

**Keywords:** high-throughput bioprinting, nasal epithelium, nasal epithelial cells, tissue models, infection

## Abstract

Progenitor human nasal epithelial cells (hNECs) are an essential cell source for the reconstruction of the respiratory pseudostratified columnar epithelium composed of multiple cell types in the context of infection studies and disease modeling. Hitherto, manual seeding has been the dominant method for creating nasal epithelial tissue models through biofabrication. However, this approach has limitations in terms of achieving the intricate three-dimensional (3D) structure of the natural nasal epithelium. 3D bioprinting has been utilized to reconstruct various epithelial tissue models, such as cutaneous, intestinal, alveolar, and bronchial epithelium, but there has been no attempt to use of 3D bioprinting technologies for reconstruction of the nasal epithelium. In this study, for the first time, we demonstrate the reconstruction of the nasal epithelium with the use of primary hNECs deposited on Transwell inserts via droplet-based bioprinting (DBB), which enabled high-throughput fabrication of the nasal epithelium in Transwell inserts of 24-well plates. DBB of progenitor hNECs ranging from one-tenth to one-half of the cell seeding density employed during the conventional cell seeding approach enabled a high degree of differentiation with the presence of cilia and tight-junctions over a 4 weeks air–liquid interface culture. Single cell RNA sequencing of these cultures identified five major epithelial cells populations, including basal, suprabasal, goblet, club, and ciliated cells. These cultures recapitulated the pseudostratified columnar epithelial architecture present in the native nasal epithelium and were permissive to respiratory virus infection. These results denote the potential of 3D bioprinting for high-throughput fabrication of nasal epithelial tissue models not only for infection studies but also for other purposes, such as disease modeling, immunological studies, and drug screening.

## 1. Introduction

The nasal epithelium is composed primarily of pseudostratified columnar epithelial cells containing basal, ciliary, non-ciliary mucus-secreting goblet cells, which play a crucial role in the physiological and immunological functions of the nasal cavity [1]. These cells are the first barrier against substances that are inhaled in daily life, such as allergens, viral, bacterial and fungal pathogens [1–3], and are responsible for [1–3] mucociliary clearance led by a wave action removing foreign bodies and preserving the homeostasis of the nasal cavity [4, 5]. Tight junctions between nasal epithelial cells play a crucial role in maintaining the integrity of the nasal epithelial barrier by sealing the spaces between adjacent epithelial cells and preventing the passage of molecules and pathogens through the paracellular space [6, 7]. These junctions are made up of transmembrane proteins such as claudins, occludins, and junctional adhesion molecules that interact with each other and with the cytoskeleton to form a stable barrier. The expression and localization of these proteins can be altered by different physiological and pathological stimuli, such as inflammation and allergens. This can lead to changes in barrier function, increased permeability of the epithelium, and the development of inflammatory conditions [7–9].

The use of bronchial epithelial cells obtained from brush biopsies has been a common established method for fabrication of pulmonary models in the literature [10]; however, these cells may not be representative of healthy epithelium due to surrounding inflammation, disease, or possible deformation in the three-dimensional (3D) epithelial structure [11, 12]. An alternative source of epithelial cells is from the nasal cavity, which can be obtained through less invasive procedures. The use of freshly isolated primary airway cells or commercially available fully-differentiated respiratory epithelial cell cultures can be costly and limit the variety of donors available [13]. Therefore, *in-vitro* culture of functional respiratory epithelium is crucial in current respiratory tissue engineering approaches to overcome these limitations [14]. Recently, respiratory epithelial *in-vitro* systems have become an alternative to expensive, labor-intensive animal studies, which also raise ethical issues, regardless of their relevance to human physiology [15, 16]. These *in-vitro* systems utilize a wide range of cell sources and cultivation protocols allowing the investigation of respiratory diseases and the effects of various environmental factors on the airways [17, 18]. Although immortalized cell lines have the potential to overcome limitations of primary cell cultures, they may lack complete differentiation and the ability to generate ciliated cells. Papazian *et al* argued that *in-vitro* differentiation of primary human respiratory epithelial cells may be the most suitable option for studying the airway epithelium despite of its complex and time-consuming process [19, 20]. To address such issues, it is important to standardize and validate methods (i.e. protocols, cells, scaffolds, and media) with inter-laboratory reproducibility, ultimately, for replacing animal testing.

Bioprinting is a rapidly growing field that uses the basics of 3D printing technologies to create living structures composed of cells and biomaterials [21–23]. One of the major purposes of bioprinting is to create functional tissue models that can be used for research, drug discovery, and eventually, therapeutic applications. Thus, bioprinting has been used to produce various types of epithelial tissue models such as skin [24], intestinal [25, 26], bronchial epithelium [27] and more recently, alveolar epithelium [28, 29]. Nevertheless, to date, researchers conventionally use manual cell seeding to develop differentiated cultures of the nasal epithelium [30]. In addition, various other techniques, such as microfluidic channels with a bioreactor [30], spheroid [31, 32] and hanging drop culture [33, 34], have been used to reconstitute the nasal epithelium composed of multiple cell types. However, manual cell seeding is a slow process, lacks throughput and does not yield uniform tight junctions, in which 3D bioprinting can overcome such limitations. To the best of our knowledge, bioprinting has not been reported to reconstruct the nasal epithelium. Bioprinting can facilitate homogeneous cell distribution with high accuracy in a high-throughput manner for investigating nasal disorders and diseases including but not limited to sinusitis, asthma, and viral infection.

In this study, we present the first-time demonstration of nasal epithelial reconstitution via droplet-based bioprinting (DBB) of progenitor human nasal epithelial cells (hNECs). To elaborate, DBB was employed by depositing hNEC-laden solution onto Transwell inserts in 24-well plates, in which each layer was deposited in a ‘pass’ over the surface, and the number of passes were varied from 10 to 50 to modulate the cell density. Upon confirmation of the biocompatibility, hNECs were maintained in submerged culture for 10 d to reach confluency and form tight junctions and then at the air–liquid interface (ALI) for three weeks to fully differentiate. Compared to manually seeded hNECs, bioprinted hNECs, at an even relatively lower cell density, exhibited successful differentiation into multiple cell types, formation of pseudostratified columnar epithelial architecture, and evidence of functionality (i.e. barrier function, mucus secretion, and beating cilia). Overall, the presented technique facilitated the effective fabrication of the nasal epithelial tissue in a high-throughput manner with structural and physiological characteristics like the native nasal epithelial tissue.

Hence, our study aims to investigate the potential of DBB as a novel approach for reconstructing functional nasal epithelial tissue models. Our hypothesis is that DBB of primary hNECs better facilitates the reconstitution of nasal epithelial tissue with structural and physiological characteristics closely resembling the native nasal epithelium compared to the conventional manual approach. Specifically, we postulate that DBB supports the differentiation of hNECs into multiple cell types, the formation of pseudostratified columnar epithelial architecture, and the development of functional features, including barrier function, mucus secretion, and beating cilia. Additionally, we anticipate that DBB offers advantages, such as high-throughput tissue fabrication and uniform cell distribution, overcoming the limitations associated with the manual approach. This study will not only advance our understanding of nasal epithelial biology but also provide a platform for investigating nasal disorders and diseases, such as sinusitis, asthma, and viral infections, in a controlled *in-vitro* environment.

## 2. Materials and methods

### 2.1. Harvesting of hNECs

hNECs were collected from the nasal turbinates of consenting volunteers with a brush (CytoSoft CYB-01) as approved by the Institutional Review Board (IRB) at Nationwide Children’s Hospital (IRB Numbers 16-00827 and 17–00594). Six sterile nasal brushes were inserted into the nasal turbinates of organ donors to dislodge and collect cells. The brushes were then placed in a solution of Ca^2+^/Mg^2+^ Dulbecco’s phosphate buffered saline (DPBS, Corning) supplemented with a 1X antibacterial-antifungal antibiotic mix and 10 *μ*M ROCK Inhibitor (Stemcell Technologies). Samples were stored on ice until the harvesting process. For the harvesting process, microfuge tubes were used, with 2 mm of the tip trimmed off. These tubes were then placed in a 15 ml conical tube, containing 12 ml of ice-cold Dulbecco’s Modified Eagle Medium (DMEM, Corning). The brushes were inserted into the hole in the microfuge tube and moved up and down to scrape the cells off and into DMEM. Next, cells were centrifuged for 5 min at 500 X g. To declump cellular material, a declumping solution was added, and the mixture was incubated at 37 °C in 5 min increments. During incubation, clumps were observed for breakup. To stop the reaction, fetal bovine serum (FBS) was added. The cells were then spun down and rinsed thrice. The cells were resuspended for seeding in PneumaCult Ex Plus (Stemcell Technologies) supplemented with 10 *μ*M ROCK Inhibitor and antibiotics. They were then seeded onto Type I bovine collagen (PureCol) coated wells or plates. The following day, the wells were rinsed with PBS and fed with the same medium. The cells were allowed to expand for a maximum of 10–14 d for preserving their intrinsic progenitor characteristics. This work was classified as a level 1 risk clinical study—no greater than minimal risk (pursuant under 45 Code of Federal Regulations [CFR] 46.404 and 21 CFR 50.51). Informed consent procedures followed in compliance with Nationwide Children’s Hospital Research Responsible Conduct Guidelines.

### 2.2. Manual initiation of nasal epithelial cultures

Progenitor hNECs were placed in DPBS with Ca^2+^/Mg^2+^ and stored at 4 °C until processing. They were removed from brushes and treated with a declumping solution containing PBS, dithiothreitol (3 mM), ethylenediaminetetraacetic acid (EDTA; 2 mM), and Type II collagenase (12.5 mg/100 ml) for 5–10 min at 37 °C followed by the addition of 10% FBS. Cells were plated on tissue culture dishes precoated with Type I bovine collagen (PureCol). They were expanded in bronchial epithelial cell growth media (BEGM) [35] or Ex Plus (Stem Cell Technologies) medium for 1 week before being cryopreserved. Upon thawing, the cells were plated on Type IV collagen (Sigma-Aldrich) coated Transwell inserts (Corning 3470; polyester, 6.5 mm diameter, 0.4 *µ*m pores) and used as a control group.

### 2.3. Characterization of droplets

The rheological properties of the bioprinting solution (cell medium) was analyzed using flow sweep measurements in the shear rate between 0.1 and 100 s^−1^ at room temperature (22 °C). A rheometer (MCR 302, Anton Paar, Austria) was utilized with a truncation gap of 53 *µ*m and a stainless-steel cone-plate that has 25 mm diameter and 1° cone angle.

To measure the density, the mass of 100 *μ*l cell medium was measured using a balance (Mettler-Toledo International), and the density was calculated by the mass divided by the volume. A customized droplet-based bioprinter (jetlab^®^ 4, MicroFab Technologies Inc.) connected with a micro-valve dispensing device (The Lee Company, Cat. No. INKX0517500A) and a removable nozzle (250 *μ*m inner diameter, The Lee Company, Cat. No. INZA3100914K) was used to dispense 1000 droplets of cell medium. The average individual volume of droplets was obtained by the mass divided by the density. Using an in-built camera, the velocity of droplets was recorded and evaluated using ImageJ software (National Institutes of Health, Bethesda, MD). Surface tension was measured using a Ramé-hart 260 Automated Goniometer/Tensiometers (Ramé-hart Instrument Co.).

### 2.4. Droplet behavior for DBB

To characterize printing performance of the cell medium, the map of dimensionless parameters was obtained by Ohnesorge (Oh), Weber (We), Reynolds (Re), and Froude (Fr) number as following equations according to a previous work [36]:

\begin{equation*}{\text{Oh}} = \frac{{\sqrt {{\text{We}}} }}{{{\text{Re}}}} = \frac{\eta }{{\sqrt {\gamma \rho L} }} = \frac{1}{Z}\end{equation*}

\begin{equation*}{\text{We}} = \frac{{\rho {U^2}L}}{\gamma }\end{equation*}

\begin{equation*}{\text{Re}} = \frac{{\rho UL}}{\eta }\end{equation*}

\begin{equation*}{\text{Fr}} = \frac{U}{{\sqrt {gL} }}\end{equation*}

where $\eta $ is the viscosity of the cell medium, *γ* is the surface tension of the cell medium, $\rho $ is the density of the cell medium, $L$ is the diameter of nozzle orifice, $U$ is the velocity of droplets, and $g$ is acceleration of gravity. In addition, Bond number and splashing parameters were obtained according to a previous study [37] as below:

\begin{equation*}{B_0} = \frac{{pg{L^2}}}{\gamma }\end{equation*}

\begin{equation*}{K_{{\text{sp}}}} = \sqrt {{\text{We}}\sqrt {{\text{Re}}} } \end{equation*}

where $\rho $ is the density of the cell medium, $g$ is the acceleration due to gravity, $L$ is the characteristic length, *γ* is the surface tension of the cell medium, ${K_{{\text{sp}}}}$ is the splashing parameter, ${\text{We}}$ is the Weber number, and ${\text{Re}}$ is the Reynolds number.

### 2.5. DBB of the nasal epithelial tissue

**Figure 1.**
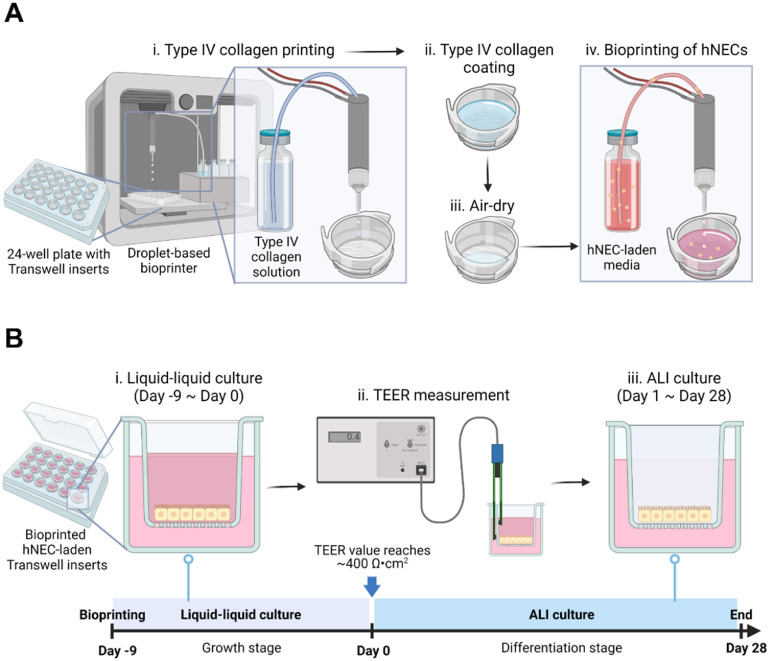
A schematic image illustrating (A) DBB of the nasal tissue model and (B) its culture along with the timeline for liquid–liquid followed by ALI culture. This figure was created using BioRender (https://biorender.com).

For a sterile environment, the customized droplet-based bioprinter was placed in a biosafety cabinet (Air Science Purair VLF36) with vertical laminar flow. A micro-valve dispensing device connected with a removable nozzle (250 *μ*m inner diameter) was used to expel solutions placed in fluid reservoirs. Then, 10 mg ml^−1^ Type IV collagen solution was printed for coating the surface of 6.5 mm Transwell inserts in 24-well plates. Here, the printing parameters were set as 5 V dwell voltage, 500 ms dwell time, 1 ms rise/fall time, 0 V echo voltage, 20 ms echo time, 100 Hz frequency, and 126 kPa pneumatic pressure. After air-drying the collagen-coated inserts in the biosafety cabinet overnight, hNEC-laden PneumaCult-ALI (Stemcell Technologies) medium (a density of 0.022 × 10^5^ cells ml^−1^) was bioprinted in a 7 mm circular shape layer by layer at room temperature (22 °C), in which a single layer was referred to as a ‘pass’ as shown in figure 1(A). The hNEC-laden structures were bioprinted in 10, 20, 30, 40, and 50 passes to compare with manually seeded hNECs to explore the role of cell seeding density on the differentiation of hNECs and formation of the nasal epithelium. During our experiments, approximately 2.2 × 10^5^ cells were seeded per insert for the manual seeding group while 0.22, 0.44, 0.66, 0.88 and 1.1 × 10^5^ were bioprinted per insert for the 10-, 20-, 30-, 40- and 50-pass group, which took ∼2, 4, 6, 8 and 10 min respectively (table 1) (video S1).

**Table 1.** Cell numbers used for manual seeding and DBB.

	Cell number per insert	Passes	Cell number per insert
Manual seeding	2.2 × 10^5^ cells	10	0.22 × 10^5^ cells
		20	0.44 × 10^5^ cells
		30	0.66 × 10^5^ cells
		40	0.88 × 10^5^ cells
		50	1.1 × 10^5^ cells

### 2.6. ALI culture of hNECs

Bioprinted and manually prepared Transwell inserts on 24-well plates were cultured for 37 d as shown in figure 1(B). The media consisted of PneumaCult-ALI basal medium (Stemcell Technologies) supplemented with PneumaCult-ALI 10× supplement, PneumaCult-ALI Maintenance Supplement (100×), Hydrocortisone stock solution, Heparin solution with the Rock Inhibitor (all from Stemcell Technologies) were mixed and added to both the apical (0.2 ml) and basal (0.65 ml) compartments. The samples were submerged for the first 10 d, which were referred as Day −9 to Day 0 (figure 1(B)). On Day 0, the apical culture medium was removed from the ALI culture and the basal chamber medium was replaced with the same PneumaCult ALI media but lacking the Rock Inhibitor. This medium was replaced every 2 d until Day 28. Excess mucus was removed from the apical surface by washing the cells once with DPBS (1X; Corning) as required but at least once per week to prevent excessive mucus accumulation.

### 2.7. Cell viability analysis

After DBB, cell viability was measured using LIVE/DEAD staining (Invitrogen, CAT: R37601) before cell differentiation at a day after DBB (Day −8) and a week later (Day −2). A staining solution was prepared by mixing (0.15 mM) and ethidiumhomodimer-1 (2 mM) and added to the apical surface of each culture, which was placed in the incubator with 5% CO_2_ at 37 °C for 30 min. The stained cells were imaged using an Axio Zoom fluorescent microscope (Zeiss). The percentage (%) cell viability was calculated by dividing the number of live cells by the total number of cells and multiplying by 100.

### 2.8. Scanning electron microscopy (SEM)

The samples were rinsed with PBS twice, then promptly fixed using a solution containing 2% glutaraldehyde buffer (Sigma-Aldrich) for 4 h at room temperature. The fixed specimens were subsequently dehydrated using a graded series of ethanol, and critically dried using critical point dryer (Leica CPD300). Standard specimen mounts were utilized to mount the dried specimens, which were then sputter coated with a layer of gold/palladium. Finally, the specimens were examined using a scanning electron microscope (Zeiss SIGMA) operating at an accelerating voltage of 2 kV.

### 2.9. Immunofluorescent staining

The variations in Zonula Occludens-1 (ZO-1), cilia expression and mucus positivity were examined using immunofluorescent imaging. hNECs were being fixed with 4% paraformaldehyde (PFA) overnight at 4 °C. They were permeabilized with 0.1% Triton X-100 (Sigma-Aldrich) in PBS for 10 min, followed by blocking solution composed of: 10% normal goat serum (Abcam), 1% bovine serum albumin BSA (Research Products International), 0.3 M glycine (Sigma-Aldrich) and 0.1% Tween 20 (Sigma-Aldrich) in 10 ml DPBS (Corning) for 1 h at room temperature. ZO-1 monoclonal antibody (Invitrogen), Alpha Tubulin (TUBA4A) mouse monoclonal antibody (Origene) and MUC5AC monoclonal antibody (Invitrogen) were each diluted to 5 mg ml^−1^ in blocking solution. The samples were incubated with ZO-1, TUBB4A and MUC5AC overnight at 4 °C and then incubated with Alexa 488-F (ab’)2 fragment of goat anti-mouse IgG (Invitrogen) for 2 h at room temperature. At the same time, nuclei were stained with 4ʹ,6-diamidino-2-phenylindole (DAPI) solution (1 mg ml^−1^) for 2 h at room temperature. The cells were thoroughly washed three times for a total of 3 min between each procedure. Images were captured using a confocal microscope (Zeiss LSM880).

ALI samples were then characterized with four major epithelial cell markers, namely basal, goblet, club, and ciliated cells. They were embedded in OCT (Sakura Finetek) and snap frozen in liquid nitrogen. The frozen samples were cut at 8 *µ*m thickness, air dried on Superfrost plus slides, then fixed with 4% PFA (15 min) and permeabilized with 1X PBS/0.1% Triton-X-100 (15 min). Tissue sections were treated with Fc Receptor Blocker, followed by Background Buster (Innovex Bioscience). The sections were stained with appropriate primary antibodies for 1 h followed by secondary antibodies for 30 min at room temperature in 1X PBS/5% BSA/0.05% saponin (table S1). For panels including pure and conjugated antibodies, an additional step of blocking with 1/20 normal mouse serum in 1X PBS (15 min) was used (table S1). Antibody validations were performed using isotype controls. Finally, sections were counterstained with 1 mg ml^−1^ of DAPI and 0.1 nmol units/ml of Phalloidin ATTO647N and mounted with Fluoromount-G (Thermo Fisher Scientific). The slides were acquired using a confocal (Leica SP8) (for high resolution images) or widefield microscope (Leica Thunder) (for histocytometry quantification based on immunofluorescent scans). Microscope acquisition was performed using LAS X software (Leica) and then image analysis was conducted using Imaris software (Oxford Instruments) (Bitplane).

### 2.10. ZO-1 quantitative analysis

With the obtained ZO-1 images, ZO-1-to-ZO-1 distance was quantitatively evaluated to represent how dense tight junctions were formed for different groups at Days 14 and 28. Using ImageJ Fiji software (National Institutes of Health), a single line was drawn on the fluorescent images to acquire the intensity profile. Then, peaks of the intensity profile were identified. Subsequently, the position of each peak was converted to the actual scale in *μ*m and the distance between two peaks was measured.

### 2.11. Quantification of hNEC differentiation markers

After acquiring cilia and MUC5AC images, the area of fluorescence was quantified using ImageJ Fiji. For the cilia area, a region of 10^5^ mm^2^ was selected and analyzed to represent the area in absolute value, and for the MUC5AC area, the area was represented as a percentage.

### 2.12. Transepithelial electrical resistance (TEER) measurements

TEER values were measured with a STX2/chopstick electrode across the epithelial layer in Transwell inserts using an EVOM2 epithelial voltage meter (World Precision Instruments). According to the manufacturer’s instructions, electrodes were sterilized and adjusted before the measurement. To eliminate excessive mucus, cells were manually washed once with pre-warmed DPBS prior to TEER measurements. Subsequently, pre-equilibrated ALI medium (Stemcell Technologies) was added to the apical (0.25 ml) and basolateral (1 ml) chambers. Before reading TEER values, the monolayers were given time to reach a stable potential for ∼5 min inside the incubator. The sample resistance was calculated by subtracting the values from the cell-free Transwell insert’s resistance. Resistance of each Transwell insert in units of Ω was calculated using a previously described technique [38].

### 2.13. Calculation of ciliary beating frequency (CBF)

After 4 weeks of ALI culture, ciliary beating of hNECs was examined using a recently described technique [39]. The inserts were washed three times with prewarmed DPBS to eliminate the mucus. CBF was recorded as a series of images using the Axio Zoom microscope. The images were taken at a sample interval of 1 ms, a frame rate of 100 frames/s and 40× microscope magnification. A series of at least 512 or 1024 images was captured over a period of 10 s. For subsequent retrieval and analysis, each set of frame-by-frame photos was saved in a TIF type file. Three randomly chosen areas of a single insert were captured. Over the recording time, the pixel intensities of a particular area of interest (ROI) were collected, and the data were used for fast Fourier transformation. Using equation (7), the CBF was determined by counting a certain number of distinct cilia beat cycles:

\begin{equation*}{\text{CBF}} = \frac{{{\text{Frame rate }}\left( {{\text{number of frames}}/{\text{sec}}} \right)}}{{5{ }\left( {{\text{frames elapsed for }}5{\text{ ciliary beat cycles}}} \right){ } \times { }5{ }\left( {{\text{conversion per beat cycle}}} \right)}}.\end{equation*}

### 2.14. Permeability study

The permeability study was performed for all groups using a 4 kDa fluorescein isothiocyanate (FITC) Dextran marker (Sigma-Aldrich, CAT:46944-100MG-F). Dextran permeability assay was performed for samples at Day 28. Dextran (10 mg ml^−1^) was applied to the apical part of Transwell inserts for cell-laden and blank samples and then all inserts were incubated in the incubator. Media was collected from the basal compartment every 60 min and loaded into 96-well plates (Greiner Bio) for 3 h. Using a microtiter plate reader (BioTek Instruments Inc.), Dextran content in the base samples were calculated using a 492 nm-excitation wavelength.

### 2.15. Single-cell RNA sequencing (scRNA-seq) dissociation

Inserts were washed with PBS, both apically and basally. They were then incubated twice with 0.05% trypsin/EDTA, apically and basally. 2% FBS and 2 mM EDTA in PBS was used to inactivate trypsin. Following each incubation with trypsin, cells were collected by gently pipetting up and down without scratching the membrane with the pipette tip. Cells were washed with PBS and then incubated in a Dispase I/DNase I solution. The samples were then filtered through a pre-wetted 30 mm cell strainer. Single cells were washed and suspended in PBS containing 0.04% BSA and immediately processed as follows. Cell viability was assessed on a Countess II automated cell counter (ThermoFisher) and up to 10 000 cells were loaded onto a single lane of a 10X Chromium X. Single cell capture, barcoding and library preparation were performed using the 10X Chromium platform version 3.1 NEXTGEMchemistry and according to the manufacturer’s protocol (#CG000388). cDNA and libraries were checked for quality on Agilent 4200 Tapestation and ThermoFisher Qubit Fluorometer and quantified by KAPA qPCR. The bioprinted insert was sequenced at 16.6% of an Illumina NovaSeq 6000 S4 v1.5 200 cycle flow cell lane, targeting 5000 barcoded cells with an average sequencing depth of 80 000 read pairs per cell. The manually seeded insert was sequenced at 8.3% of an Illumina NovaSeq 6000 S4 v1.5 200 cycle flow cell lane with an, targeting 2000 barcoded cells with an average sequencing depth of 70 000 read pairs per cell. Illumina base call files for all libraries were converted to FASTQs using bcl2fastq v2.20.0.422 (Illumina) and FASTQ files associated with the gene expression libraries were aligned to the GRCh38 reference assembly with v32 annotations from GENCODE (10xGenomics GRCh38 reference 2020-A) using the version 6.1.2 Cell Ranger multi pipeline (10xGenomics). Raw data are available from https://github.com/ohlab.

### 2.16. scRNA-seq analysis

Seurat (version 4.2.1) [40] was performed in R and was applied to all datasets. For downstream analyses, we kept cells that met the following filtering criteria per condition: >2500 genes/cell, >80% number of genes per unique molecular identifier for each cell, and <20% mitochondrial gene expression. R package scDblFinder (Version 3.17) [41] was used to identify doublets and keep only singlets. Using Seurat, the manually seeded and bioprinted inserts were merged, mitochondrial gene expression was regressed out using SCTransform v2, and the samples were integrated. For clustering, we used the functions FindNeighbors and FindClusters, which implement Shared Nearest Neighbor modularity optimization-based clustering algorithm on 30 principal components (PCs) with a resolution of 0.1. Nonlinear dimensionality reduction methods, namely t-distributed stochastic neighbor embedding and uniform manifold approximation and projection (UMAP), were applied to the scaled matrix for visualization of cells in two-dimensional space using 30 PCs. Specific marker genes were obtained from previously published studies [42, 43] and visualized using FeaturePlot and DimPlot.

### 2.17. Viral infection

Influenza virus (PR8-GFP, with GFP fused to NS1; A/PR8/34 (H1N1) [44]) was obtained from Dr Adolfo García-Sastre (Icahn School of Medicine at Mount Sinai). Infectious stocks were prepared on Madin–Darby canine kidney (MDCK) cells and supernatants were centrifuged and filtered (0.45 mm). Viral titers were determined by plaque assay on MDCK cells. Aliquots were stored in a secured −80 °C freezer until further use.

Prior to infection, the apical surface of the ALI cultures was washed 1–3 times with PBS (1X) for 15 min at 37 °C to remove the mucous. Then, the ALI samples were apically exposed to 25 ml of virus solution. The virus was diluted in Pneumacult ALI media serum-free for a final concentration of 2.5 × 10^5^ pfu PR8-GFP. Following 24 h incubation, 100 ml of PBS was added to the apical side of the ALI cultures for 15 min at 37 °C and harvested in an Eppendorf tube. Flow cytometric monitoring of virus infection was analyzed using recently described studies [45, 46].

### 2.18. Histocytometry

To proceed to histocytometry, we adopted the Germain methodology [47]. Briefly, three consecutive sections, 8 mm thick, were acquired and the intensity mean for cell populations positive for DAPI (*Y* axis) and viral GFP (*X* axis) were generated in Imaris 9.7 (Oxford Instruments) using the Channel Arithmetics Xtension prior to running surface creation to identify DAPI-GFP cells in images. Statistics were exported for each surface and imported into FlowJo v10.3 (BD Bioscience) for image analysis.

### 2.19. Statistical analysis

The data were displayed by using GraphPad Prism 8 (GraphPad Software) and statistical analysis was carried out using SPSS 28 software (SPSS, Inc.). One-way ANOVA with post-hoc Tukey tests was used for comparison of the differences among multiple groups, which were indicated as *p < 0.05, **p < 0.01, and ***p < 0.001.

## 3. Results

### 3.1. DBB of the nasal epithelial tissue

To verify the printing performance of cell medium via DBB, dimensionless numbers, including Reynolds, Weber, Ohnesorge, and Bond number, were used to describe the droplet behavior on solid-to-liquid and liquid-to-liquid interface [48–51]. To begin, the Bond number indicating the ratio of gravitational force to surface tension force was <1 so that gravitational impact was negligible. It denoted that droplet spreading on solid substrates was driven by inertial or capillary force. On the other hand, initial spreading of droplets was caused by impact pressure but it was not significant owing to inertial oscillations (figure S1(A)). The aim of DBB was to cover the surface of Transwell inserts (6.5 mm diameter), and the bioprinted cell medium successfully covered the hydrophilic surface after the first pass in a 7 mm circular shape. Then, from the second pass, the droplet behavior could be explained with the liquid-to-liquid interface (figure S1(B)) [52]. The droplet was expelled at the terminal velocity, but its splashing was not significant. The splashing parameter (${K_{{\text{sp}}}}$) was calculated as 5.1, and ${K_{{\text{sp}}}}$ < 57.7 is known to not cause significant splashing [49]. Based on these results, the cell medium (bioink) allowed stable deposition on both solid and liquid interface without significant splashing and revealed adequate bioprinting behavior for DBB.

**Figure 2.**
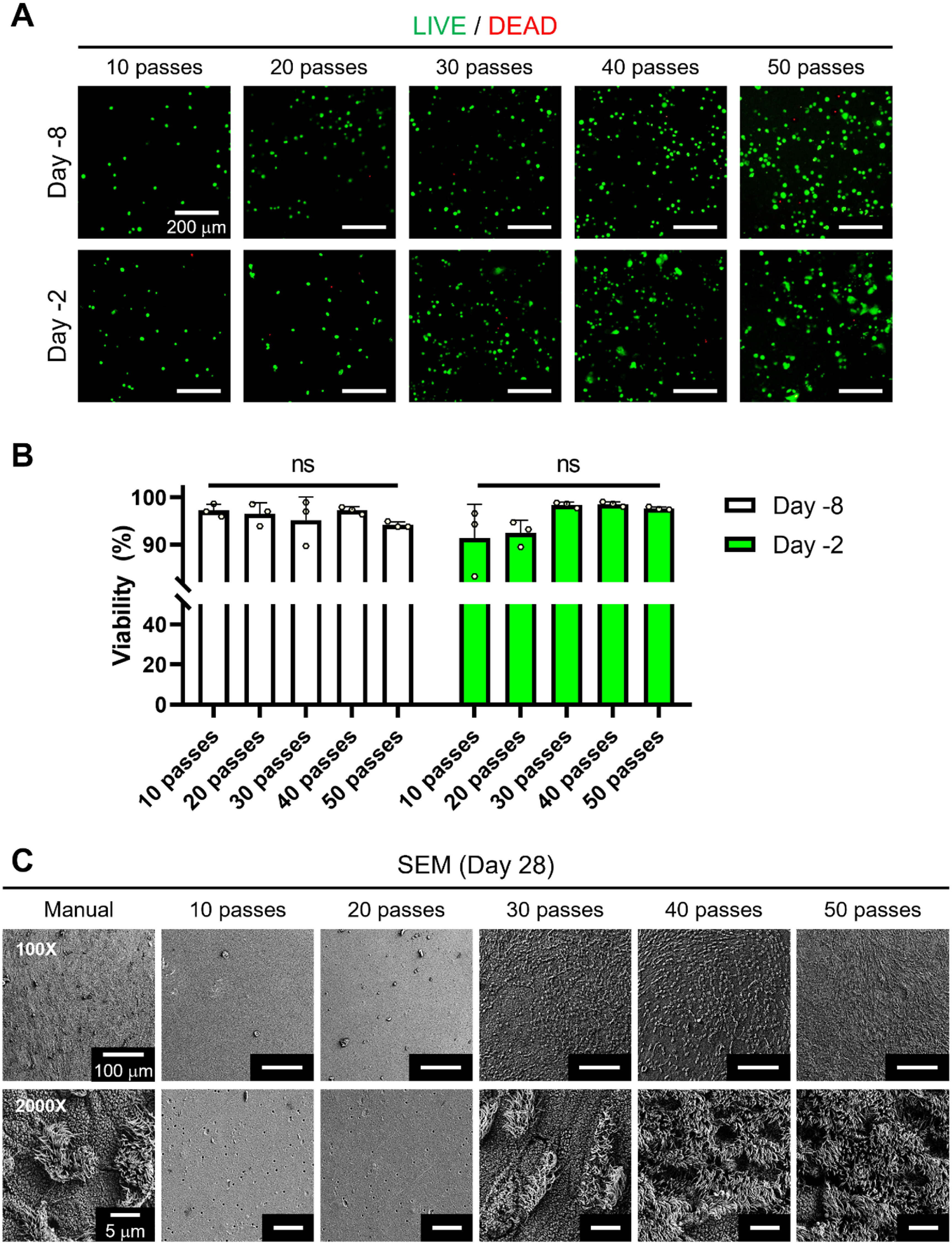
Cell viability measurements using the LIVE/DEAD assay. (A) Representative fluorescent images of hNECs at different densities at Day −8 and Day −2, and (B) cell viability (%) for all bioprinted groups including 10-, 20-, 30-, 40- and 50-pass groups (*n* = 3; ns denotes ‘not significant’). (**C**) SEM images of hNECs at Day 28 of ALI culture.

Next, we verified the biocompatibility of DBB using LIVE/DEAD staining, which captured live (green) and dead (red) cells at Day −8 and −2 (figure 2(A)). A day after bioprinting (Day −8), all groups showed high cell viability (over 90%), and such a viability level was maintained for a week (Day −2) after bioprinting (figure 2(B)). These results indicate that DBB was compatible with hNECs as cells were able to attach, grow, and differentiate afterwards.

After confirming the cell viability, cell growth was tracked and captured for a more comprehensive analysis. Figure S2 demonstrates optical images of hNECs at Days −9 (a day after DBB), 0 (the end of liquid–liquid culture), and 28 (the end of the differentiation process). At Day −9, hNECs were identified in all groups including manual seeding and bioprinted (with different number of pass groups). However, by the end of the liquid–liquid culture (Day 0), it was clearly shown that the cell population did not further expand for 10- and 20-pass groups. To maintain cellular activities, hNECs may require cell–cell interactions, but 10 and 20 passes did not provide the required cell density for these cell–cell interactions. Also, SEM images at the end of the differentiation process (Day 28) revealed no evidence of differentiation in 10- and 20-pass groups (figure 2(C)). Although less prominent cilia were observed in the manual and 30-pass groups, cilia differentiation was observed with the pass number over 30. These findings indicate that, in contrast to 10- and 20-pass groups, there was an observable increase in cilia differentiation as the number of passes increased.

Hence, while other samples started to form cell layers from Day 0, 10- and 20-pass groups revealed single cell configuration until the end of differentiation process (Day 28). For the rest of the study, 10- and 20-pass groups were not considered, and experiments were carried out using the samples of manual seeding (control) and bioprinted (with the number of passes over 20) group.

**Figure 3.**
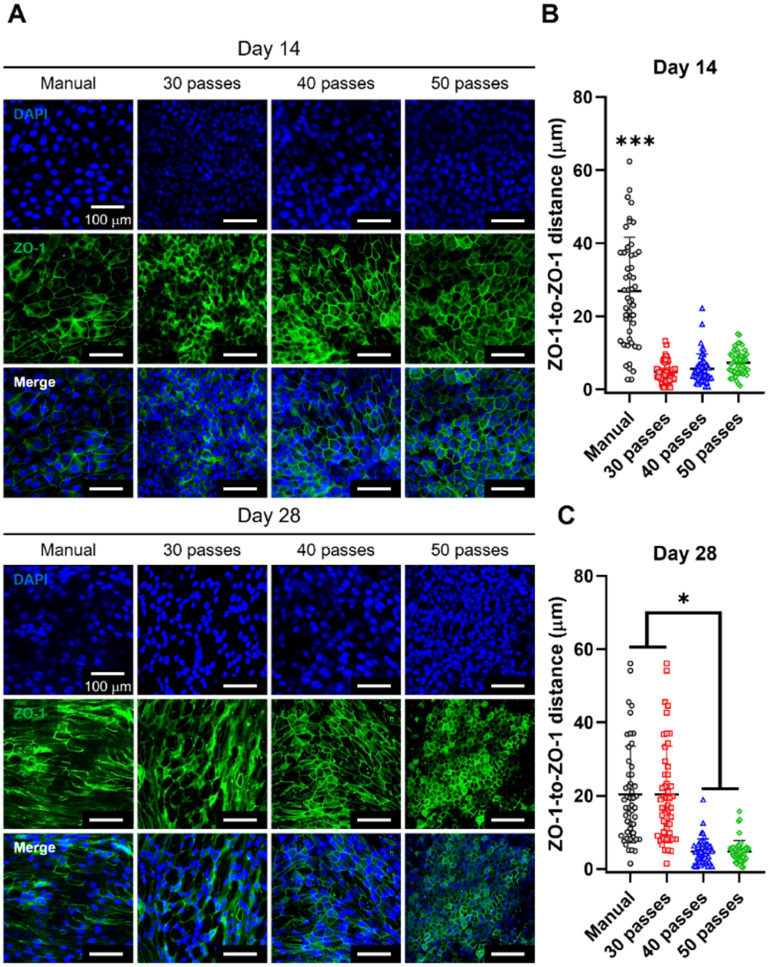
Evaluation of tight junctions in nasal epithelium using ZO-1 staining. (A) Confocal images of ZO-1 staining of hNECs in ALI culture of manual (control) and bioprinted (30-, 40- and 50-pass) groups at Weeks 2 and 4. Characterization of ZO-1 staining with respect to ZO-1-to-ZO-1 distance at Week (B) 2 and (C) 4 (*n* = 3; *p* ^*^ < 0.05 and *p* ^***^ < 0.001).

To confirm the tight junctions, ZO-1, a classical scaffold protein maintaining cell–cell adhesion found in stable tissues, was imaged (figure 3(A)), and ZO-1-to-ZO-1 distance was measured at two different time points, Day 14 (middle of the differentiation process) and Day 28 (end of the differentiation process) [53]. We observed a significant difference between the samples from manual seeding and bioprinted groups at Day 14 as shown in figure 3(B). At Day 28, when the differentiation process was completed, 30-pass and manual seeding groups became similar but there was a significant difference between manual seeding and 40- and 50-pass groups (figure 3(C)). Overall, we showed that tight junctions can be modulated by the number of passes and distributed more uniformly when DBB was applied.

**Figure 4.**
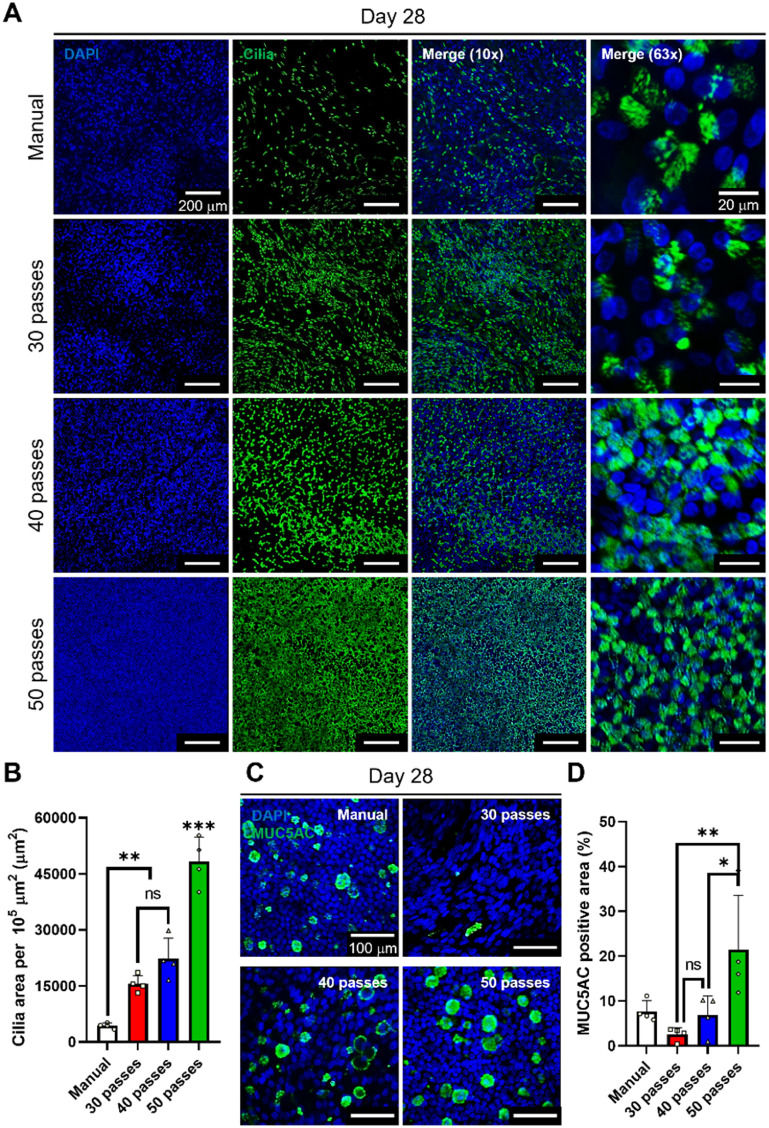
Evaluation of cilia formation and mucus secretion of nasal epithelium fabricated via manual seeding or bioprinting using a-Tubulin (cilia) and MUC5AC staining. (A) Confocal images of cilia staining of hNECs at Day 28 of ALI culture. (B) Characterization of cilia expression using cilia area per 10^5^ *μ*m^2^ at Day 28 of ALI culture. (C) Confocal images showing MUC5AC staining of hNECs at Day 28 of ALI culture and (D) related characterization of mucus production using MUC5AC positive areas (%) (*n* = 3; *p* ^*^ < 0.05, *p* ^**^ < 0.01 and *p* ^***^ < 0.001; ns denotes ‘not significant’).

Since the differentiation process was completed at Day 28, cilia and MUC5AC positive area were evaluated (figure 4). According to our calculations, the ratio of cilia per 10^5^ mm^2^ in the manual seeding group was much less compared to that in bioprinted groups (figures 4(A) and (B)). This leads us to conclude that the bioprinted groups had a higher density of ciliated cells implying accelerated cell development and differentiation. On the other hand, there was a significant difference between 30- and 50-pass groups in terms of MUC5AC staining (figures 4(C) and (D)). These results indicated that the 50-pass group was more differentiated in terms of the presence of goblet cells.

### 3.2. Functional analysis of the bioprinted nasal epithelium

**Figure 5.**
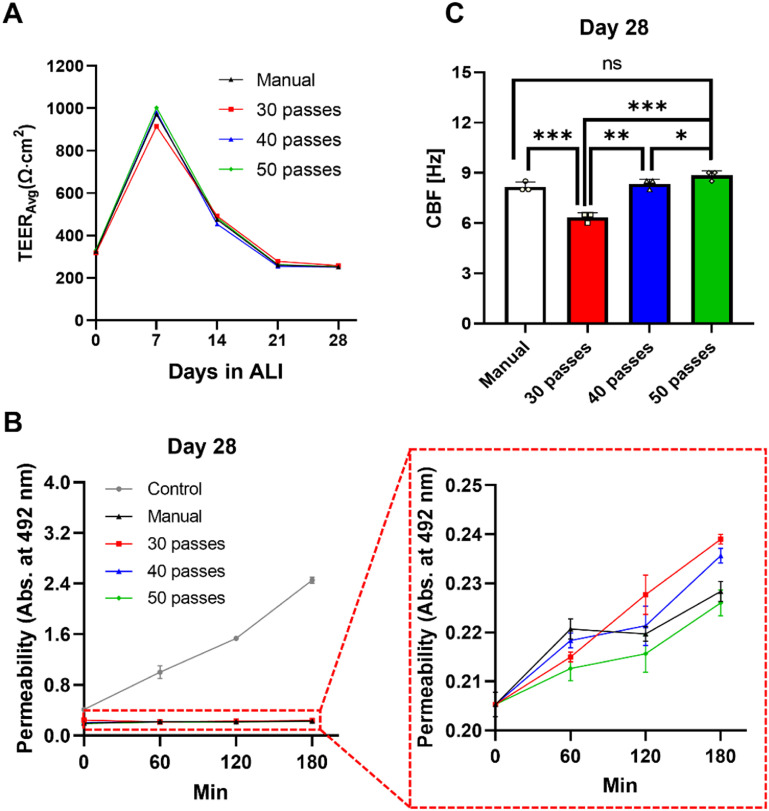
Functional analysis of nasal epithelial tissues, including manual and bioprinted (30-, 40- and 50-pass) groups. (A) Measurement of TEER values during the 28 d differentiation. (B) Comparison of Dextran permeability and (C) CBF at Day 28 (*n* = 3; *p* ^*^ < 0.05, *p* ^**^ < 0.01 and *p* ^***^ < 0.001; ns denotes ‘not significant’).

In this study, to verify the barrier function of bioprinted nasal epithelium, TEER measurements were performed for the first 4 weeks of ALI culture (figure 5(A)). The mean TEER value after a week in ALI was 1003 Ω × cm^2^ for the 50-pass group was higher than the average TEER of 971 Ω × cm^2^ for the manual group (*p* = 0.0003). After 28 d of differentiation under ALI, the TEER value was 252 Ω × cm^2^ for the manual group and 255 Ω × cm^2^ for the 50-pass group. Overall, all groups exhibited a similar TEER trend over time and the TEER measurements at Day 28 were similar. In addition, the tightness of the epithelium was confirmed by a permeability study (figure 5(B)). The flow of 4 kDa FITC-labeled Dextran from the upper chamber to the lower chamber was significantly reduced for the 28 d ALI cultured samples compared to the cell-free synthetic Transwell membranes (control). The corresponding reduction in flux rate was similar among manual, 30-, 40- and 50-pass groups, but decreased as the number of passes increased. The Dextran influx results supported the findings with TEER measurements that our samples allowed the formation of a tight epithelium under the same differentiation conditions.

A functional nasal epithelium *in-vivo* is characterized by synchronized beating of cilia. To confirm the ciliary function of ALI cultured hNECs at Day 28, CBF was determined by microscopic recording combined with image analysis (figure 5(C)). The median CBF of the 50-pass group was 8.83 Hz, which was in a comparable range with respect to the manual group (8.17 Hz). The median CBF for the 40-pass group was 8.34 Hz while the median CBF for the 30-pass group was 6.34 Hz. Overall, the CBF values increased as the number of passes increased for the bioprinted groups (video S2).

As the functional analysis revealed similar TEER and permeability measurements for all bioprinted groups, we proceed with the 30-pass group for the rest of the study.

### 3.3. Histological and scRNA-seq analysis

**Figure 6.**
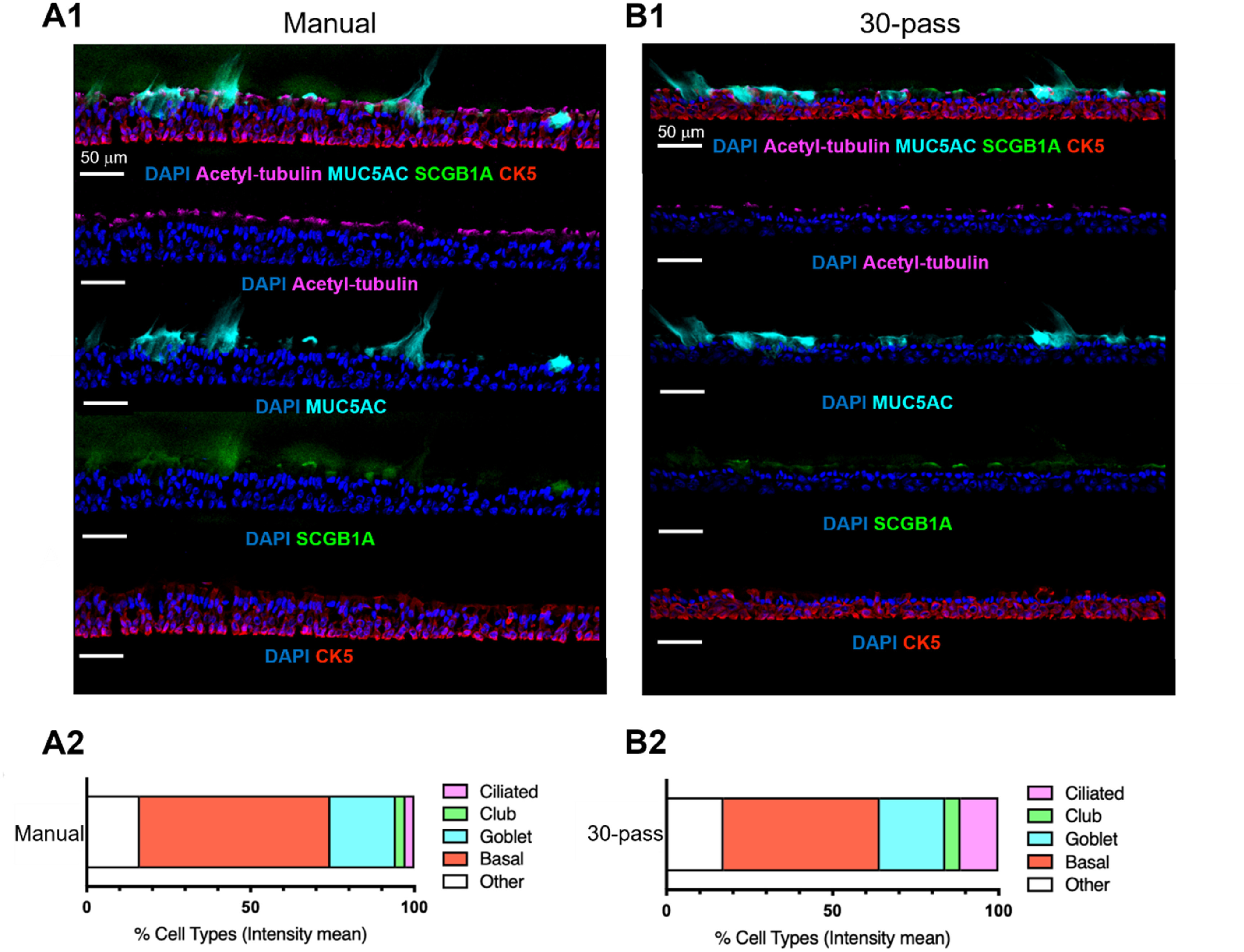
Cellular composition of manual seeding and bioprinted (30-pass) nasal epithelium. (A1) and (B1) and representative immunofluorescent images of differentiated hNECs characterized by major epithelial cell markers: basal (CK5, red), goblet (MUC5AC, cyan), club (SCGB1A1, green) and ciliated cells (acetylated *α*-tubulin, magenta) and nuclei (DAPI, blue). (A2) and (B2). Quantification of epithelial cell types in nasal ALI cultures by histocytometry (*n* = 1).

Next, we performed histological study using immunofluorescence imaging for the manual seeding (control) and the 30-pass group. We used cell-type specific markers to visualize and quantify the presence of major epithelial cell types (figures 6(A) and (B)). Bioprinted tissues effectively recapitulated the *in-vivo* upper airway pseudostratified ciliated columnar epithelial architecture with the presence of ciliated, club, goblet and basal cells [54, 55]. These data indicated that hNECs were well-differentiated with mainly consisting of basal cells. The presence of functional mucus-producing cells was further supported by the staining of mucus-containing glycoproteins with MUC5AC. The presence of functional mucus-producing cells was further supported by the staining of mucus-containing glycoproteins with MUC5AC. A high amount of mucus staining was observed in 28 d ALI culture as the differentiation was attained at the highest level at Day 28. Interestingly, the 30-pass bioprinted group presented more ciliated cells, probably due to a more homogenous and accurate distribution of cells during the high-throughput deposition.

**Figure 7.**
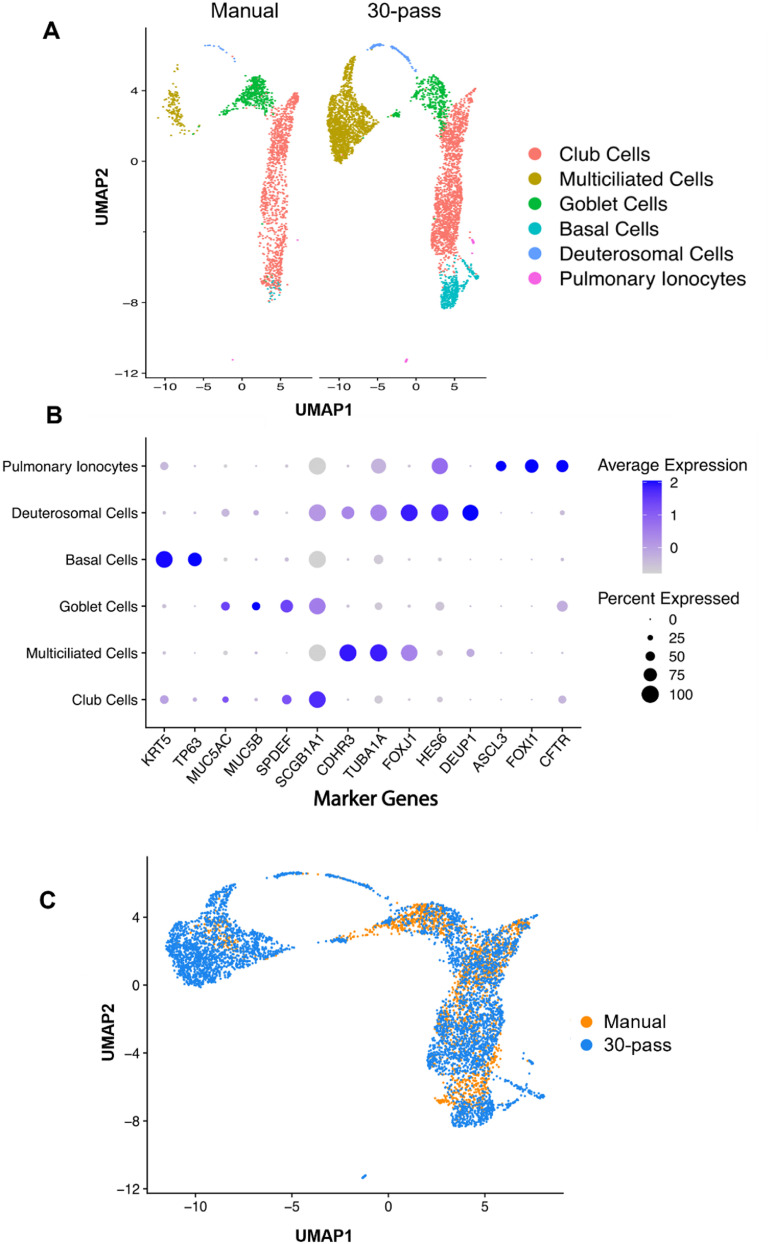
Defining bioprinted nasal cell populations using scRNA-seq. (A) UMAP plot visualization of single cells isolated from manually seeded (1829 cells) and bioprinted (4679 cells) nasal epithelial inserts that passed quality control metrics using Seurat. Putative cellular communities are presented on the right indicated in the legend. (B) Dotplot heatmap of average scaled gene expression of known nasal epithelial marker genes split by cell cluster using Seurat for merged data of manual seeded and bioprinted inserts. Size of the dot indicates percent of cells expressing that gene and the shade of purple represents the average gene expression. (C) UMAP overlay of manually seeded (orange) and bioprinted (blue) samples to show similarity of cell populations.

Next, we performed scRNA-seq analysis for the manually seeded and 30-pass bioprinted groups. We sequenced a total of 7315 cells from a bioprinted nasal insert and 2774 cells from a manually seeded insert with scRNA-seq. After performing quality control using Seurat [40] (figures S3(A) and (B)) and scDblFinder [41], we assembled RNA sequencing data from 4679 (bioprinted) and 1829 cells (manually seeded) for clustering analysis. We found six nasal tissue cell populations that were visualized by UMAP embeddings (figure 7(A)). Clusters were identified by gene expression of specific marker genes (figures 7(B) and S3(C)–S5), determining six major populations, including basal (marker genes: KRT5, TP63), deuterosomal (FOXJ1, HES6, DEUP1), club (marker gene: SCGB1A1), goblet (marker genes: MUC5AC, MUC5B, SPDEF), multiciliated (marker genes: CDHR3, TUBA1A), and pulmonary ionocyte cells (marker genes: ASCL3, FOXI1, CFTR). We compared the cell clusters between the manually seeded and 30-pass bioprinted groups (figures 7(A) and (C)). We found that, even when factoring in the increased number of cells, the bioprinted group had a more even distribution of cell types. For example, the manually seeded group had fewer basal and multiciliated cells relative to the bioprinted group (table S2). Single-cell studies of *in-vivo* human nasal epithelial tissue reported 5–7 cell types (basal, suprabasal, deuterosomal, club, tuft, pulmonary neuroendocrine, and pulmonary ionocyte cells), of which approximately half of the cells in *in-vivo* nasal epithelium were basal cells and multiciliated cells [42, 43, 56], which was better supported by the bioprinted group.

### 3.4. Viral infection of the nasal epithelium

**Figure 8.**
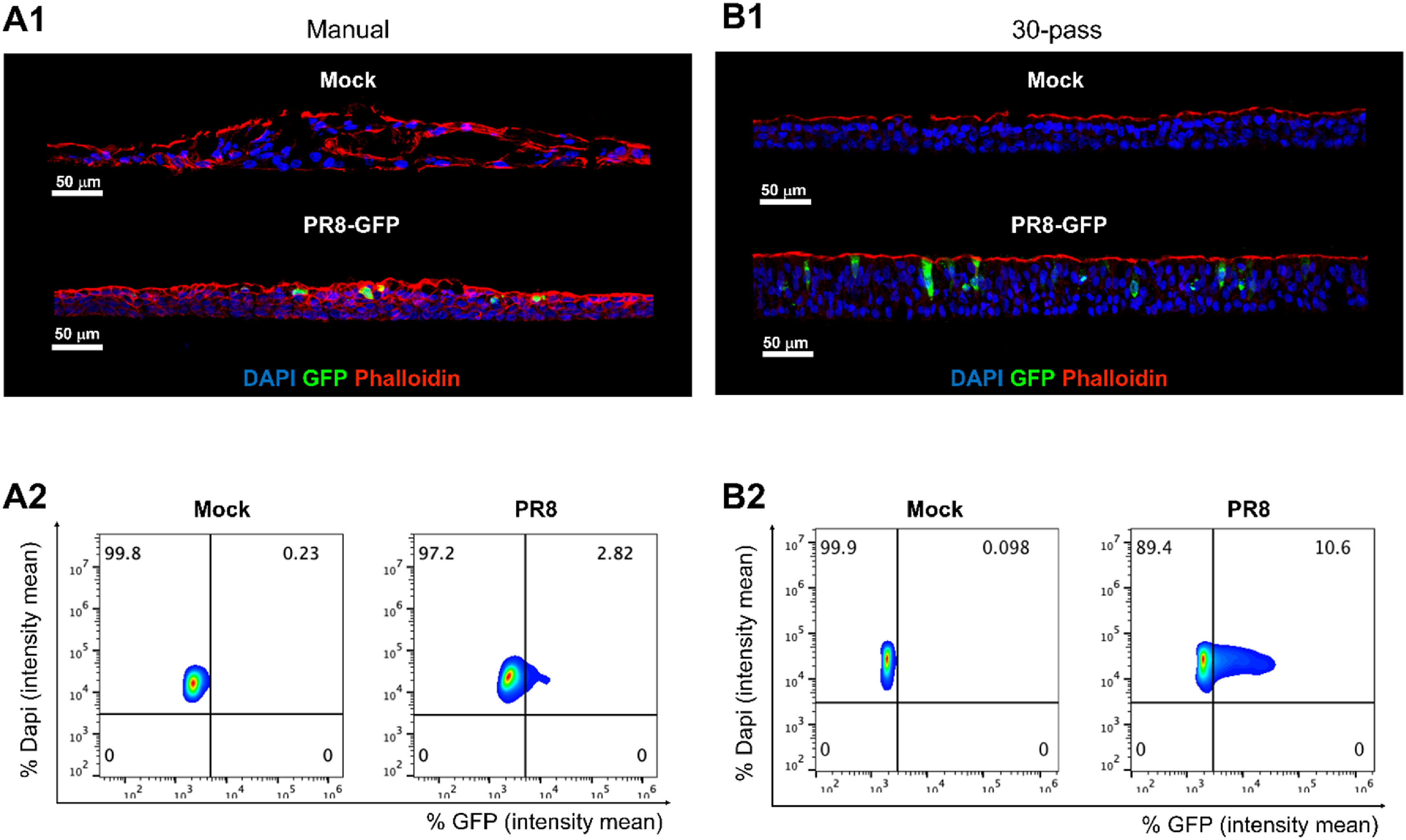
Primary human nasal epithelial ALI cultures permissive to PR8-GFP influenza infection. Representative IF images of nasal ALI from (A1) manual seeding and (B1) bioprinting (30-pass) following 24 h exposure to uninfected control (Mock) or influenza virus (2.5 × 10^5^ pfu PR8-GFP); nuclei (DAPI, blue), phalloidin (actin filament, red), and GFP expressing influenza virus to reveal effective viral replication (GFP, green). Scale bar 50 *μ*m, in white on the left corner. Representative histocytometry dot plots of (A2) manual seeding ALI and (B2) high-throughput bioprinted ALI with Mock and PR8-GFP conditions, showing the intensity mean for cell populations positive for DAPI (*Y* axis) and virus reporter GFP (*X* axis).

Lastly, we determined if these high-throughput bioprinted primary human nasal ALI cultures were permissive to respiratory viral infection. For that, we exposed ALI cultures to influenza virus strain A/PR8/34 carrying GFP (2.5 × 105 pfu) in serum-free Pneumacult ALI media, or to media without virus (uninfected control, Mock). Following 24 h of incubation, we harvested ALI cultures, then subsequently performed immunofluorescence staining. We detected the presence of virus through green fluorescent protein (GFP) and visualized the structural integrity of the cultures using Phalloidin (F-Actin) staining. The presence of GFP signal indicated active infection as GFP was a gene reporter for NS1 (nonstructural protein 1). The data demonstrated the functionality of the presented model as shown in figure 8. Interestingly, the high-throughput bioprinted ALI cultures presented an increased presence of DAPI-GFP signal (∼10.6%) by comparison to the ALI cultures from manual seeding (∼2.8%), probably due to the higher homogeneity of the bioprinted group.

## 4. Discussion

To better understand the role of respiratory epithelial cells in protecting the body and regulating the immune response, it is important to study these cells in a way that mimics the *in-vivo* situation. This can involve looking at differences among epithelial cells from diseased populations or studying the effects of external factors on these cells [57, 58]. Human airways contain many distinct types of cells, including ciliated, goblet, and basal cells, and studying these cells in an *in-vivo* like setting can provide valuable insights [59]. Using an *in vitro* model of the human nasal tissue, researchers can study specific differences in nasal epithelial cells related to different diseases, investigate the underlying mechanisms of these diseases, and control exposure to air pollutants [60–62]. Additionally, researchers can examine cell–cell interactions in a controlled environment by growing these cells on tissue culture inserts [63, 64].

In this study, we developed a method for bioprinting and investigating human nasal epithelial tissue, which has been rarely studied compared to the bronchial epithelial cultures. There are multiple advantages of studying nasal epithelial tissue instead of its bronchial counterpart [65–69]. First, nasal epithelial cells are often the first to be exposed to environmental stressors, such as pollutants or allergens, making them valuable models for studying the effects of these stressors on airway epithelial cells. Secondly, it is not always possible or appropriate to perform bronchoscopy to obtain lower airway cells in individuals with certain pre-existing diseases. Instead, the less invasive method of brushing the nasal turbinate can be used. Additionally, nasal biopsies can be done multiple times without significant side effects, making them useful for repeated studies [70–73].

There are various commercially available cell culture systems of pre-differentiated human epithelial cells, such as the EpiAirway model from MatTek, Epithelix Sárl from Switzerland, and Clonetics from Lonza, which are known to be stable (reproducible using standardized protocols and operating procedures and meeting quality standards) [74–77]. However, these systems can be expensive, and the number of samples is limited. Additionally, it can be difficult to control the characteristics of the donors, such as age, gender, and disease status, when using commercially available cell cultures. On the other hand, using freshly obtained nasal epithelial cells from superficial brush or scrape biopsies allows the researcher to control the characteristics of the donors and to recall the same volunteers for follow-up studies. Bioprinted epithelial tissue culture models are a promising alternative to the epithelial tissue models produced using manual approaches, such as aforementioned tissue models on the market. Bioprinting has several advantages compared to the manual approaches: it can be high-throughput and precise. In addition, it is a highly automated process, enabling the rapid production of large numbers of tissue constructs. This allows for high-throughput experimentation, such as large-scale drug and microbial screening [78, 79]. Furthermore, it enables precise control over the spatial arrangement of cells, which is significant for creating functional tissue structures. This is particularly useful for creating tissue models that mimic the structure and organization of native tissues [80, 81]. Besides, bioprinting allows for the homogeneous distribution of cells within a tissue construct ensuring that the cells are evenly distributed and that the tissue has a homogeneous cellular composition and tight junctions, which might be essential to create tissues with barrier function [82, 83]. Lastly, bioprinting can help with the reduction in the costs associated with manual cell culture techniques (less media, less equipment, etc) as it allows for the rapid and efficient production of primary tissue cultures [84].

The optimization of the bioprinting process was essential to ensure that the bioprinted nasal epithelial tissue was functional and mimics the structure of its native counterpart. Although there are multiple studies on hNECs and optimization of their ALI culture [13] for viral infection [85] or drug screening [86, 87], this is the first study to use bioprinting of hNECs. With this study, we have shown that bioprinting much lower number of cells with respect to the traditional manual seeding approach, results in nasal epithelial tissues with a higher degree of differentiation. Our study confirms that despite the differences in the initial cell numbers, the proportion of ciliated and goblet cells were comparable to previously reported studies. The proportion of ciliated cells in culture conditions was consistent with the proportion reported in normal, healthy human airway epithelium (50%–70%) reported previously [88]. However, the proportion of goblet cells found in our study was higher than the typical percentage found in adult human airway epithelium, which was reported to be up to 25% of cells [89]. The authors noted that the proportion of goblet cells found in that study was also higher compared to the previous studies of the authors on cultures derived from newborn and 1 year-old infants [90]. Even though the reason for this discrepancy is unclear, it may stem from donor- or age-specific factors.

Permeability data, particularly for multi-layered structures, is essential in determining the applicability of permeability values obtained from *in-vitro* experiments to clinical studies. It is important to note that various cell layers within human epithelial tissues possess distinct permeability properties, and the thickness of the culture model or tissue should be considered and controlled when comparing TEER or permeability values between different *in-vitro* models [91]. Nasal epithelial tissue, maintained for 28 d, exhibited high levels of cell shedding at the ALI interface, which may have been responsible for the increased dextran permeation. This is supported by the TEER measurements, which showed that TEER of the nasal epithelium decreased as the cells were cultured for longer periods of time. Together, these findings suggest that cell shedding at the ALI interface is likely a major contributor to the observed increase in Dextran permeation. TEER values are a measure of the integrity of the epithelial cell monolayer, which is a crucial aspect of tissue culture [38, 92]. TEER values in the presented study were higher than those reported in previous studies of human-adult [93] and pediatric nasal epithelial cultures [94]. The maximum TEER values reported in those studies were ∼400 ohm × cm^2^ after 9 d and ∼150 ohm × cm^2^ after 14 d. The values indicate that the epithelium in the current study had a higher integrity, meaning that the cells were more tightly packed and had better barrier function, than the cells reported in these earlier studies. Other than TEER, CBF plays a critical role in the mucociliary clearance process; however, the mechanisms responsible for regulating CBF are yet to be fully understood [95]. In our study, the mean CBF for all samples ranged from 6.34 to 8.83 Hz, slower than the typical CBF of healthy cilia in the human respiratory mucosa. According to the literature, the CBF of healthy cilia in the human respiratory mucosa is typically around 11–16 Hz [96, 97]. This suggests that the cilia in our samples may not be functioning as perfect as healthy cilia. The deviation of the mean CBF from the literature values is quantified by the standard deviation of 5.4 Hz, which indicates that some of the samples had CBF within the range of healthy cilia and some of them were lower. On the other hand, the results of our study pertaining to CBF values are in agreement with recent studies on human nasal epithelial cell cultures exposed to repetitive preservatives, suggesting consistency and potential applicability of the current study’s findings to understand the effects of preservatives on CBF [98, 99].

One of the advantages of the presented approach is that it allows for the creation of tissue that closely mimics the structure and function of the native nasal epithelium, which can be useful for studying the behavior of nasal epithelial cells in response to different drugs or pathogens, as well as for developing new treatments for nasal conditions such as chronic sinusitis. Indeed, the still ongoing COVID-19 pandemic has shown the emergence of *in vitro* models, such as ALI cultures, to study the response to SARS-CoV-2 virus [100, 101]. Furthermore, the evolution of the virus with the emergent variants of concern, has shown that *in vitro* ALI cultures from upper airway epithelium, nasal and bronchial, are highly permissive to SARS-CoV-2 variants [54, 55, 102, 103]. Personalized medicine is another potential application of the bioprinted nasal epithelial tissue model. By bioprinting patient-specific hNECs, it may be possible to create personalized treatments that are more effective and have fewer side effects. Despite the potential benefits, there are still challenges, such as biochemical properties, bioactivity and mechanical properties that need to be addressed before using such a system in clinical practice [104].

## 5. Conclusion

This study presents the development of a nasal epithelial tissue model using primary hNECs in a high-throughput manner via DBB. Progenitor hNECs were bioprinted with different numbers of passes ranging from 10 to 50, altering the cell density and benchmarking our results against the commonly used manual seeding approach. After printing, bioprinted hNECs showed high cell viability regardless of the pass number and we demonstrated that tight junctions, mucus secretion, and ciliary beat frequency can be modulated by the number of passes. Bioprinted hNECs resulted in a higher degree of differentiation compared to manual seeding, even though the bioprinted cell density was half or one third that of the manually seeded cell density. Five cell populations were identified in the bioprinted tissue mimicking the native nasal epithelial tissue and the trajectory analysis revealed that the bioprinted hNECs had the potential to differentiate into goblet and multiciliated cells. The presented bioprinted nasal epithelial tissue method may inspire a shift in current hNEC practices and can be used for use in various applications such as infection studies, drug testing and disease modeling.

## Data Availability

The single cell matrix is available from https://github.com/ohlab/SC2200969_Nasal30pass. Raw fastq data available upon request.
